# Using Stronge Teacher Evaluation System to Assess the Effectiveness Level of Mr. Brown as an EFL Teacher in the Mind Your Language TV Show: An Attempt to Validate a Reflective Tool to Train Preservice EFL Teachers

**DOI:** 10.3389/fpsyg.2021.648760

**Published:** 2021-10-06

**Authors:** Olusiji Lasekan

**Affiliations:** Departamento de Educación Media, Universidad Católica de Temuco, Temuco, Chile

**Keywords:** Mind Your Language, effective EFL teacher, reflective practice, TV show, teacher evaluation, teacher effectiveness

## Abstract

To develop a reflective tool for promoting teacher effectiveness among preservice EFL teachers, this article seeks to investigate the effectiveness degree of an EFL teacher as it is being depicted in a popular TV show. This is conducted using the Stronge teacher evaluation system to assess the main character’s level of effectiveness as an EFL teacher (Mr. Brown) in the Mind Your Language TV show. Drawing upon the intersection of the character’s effectiveness within the framework of characterization, representation, and perceived realism theory, a qualitative research method involving seven performance indicators of the Stronge teacher evaluation system was adopted to assess the main character. The findings showed that the character is a highly effective EFL teacher because his personal and professional characteristics are evidenced in the evaluation system’s seven performance standards. This suggests that the Show can be used as a reflective tool by preservice EFL teachers to construct and enhance their professional identities and instructional practices. This work contributes to the existing knowledge of teachers’ representation in movies and its implication in preservice EFL education by providing the first study on the representation of EFL teachers in a TV show. A future research direction is also presented.

## Introduction

The question of what constitutes an effective teacher’s personalities has been a subject of debate in the past decade (Rockoff et al., 2011; Ansari and Malik, 2013). This is owing to the belief that the effective learning of students depends on effective teaching of teachers (Alrefaee and Al-Ghamdi, 1987). Therefore, teachers need to be well aware of the objectives, goals, as well as other stakeholders’ and students’ expectations to which they are deemed to live up to (Mazandarani and Troudi, 2021). It can be observed that adjectives such as good, quality, and competent have been adopted interchangeably to describe excellent educators in several studies (Gower and Byrne, 2012; Miller, 2012; Goodwin and Kosnik, 2013). Common specific conduct and dispositions have been found among highly effective teachers. This includes the ability to use different approaches and strategies needed to improve students’ learning and achievement (Stronge et al., 2011) and possessing personal attributes such as perseverance and dedication in fighting for one’s beliefs, a willingness to take risks to achieve students’ academic goals, and pragmatism (Colker, 2008). Some experts also think that effective teachers should add instructional strategies (Harding and Parsons, 2011), classroom management (Gordon, 2012), and knowledge of the subject matter (Stronge and Tonneson, 2012) to their attributes and teaching practices. Nevertheless, considering the dynamic role of teaching and the variety of contexts in which teachers work (Lewis et al., 1999), there is no direct formula for what makes a teacher effective (Whitaker, 2013).

Over the years, the nature and purpose of teacher evaluation have evolved from a moralistic and ethical perspective that focuses on personal attributes to comprehensive learner-centered and classroom-based assessment (Ellett and Teddlie, 2003) and a system that supports the professional growth and performance accountability of teachers (Stronge et al., 2008). The latest and most widely adopted comprehensive teacher evaluation instrument is the Stronge teacher evaluation system developed from extant research related to effective teachers’ qualities (Stronge, 2012). Its effectiveness in assessing the qualities of teachers in various dimensions has been reported in different contexts (Grant et al., 2013; Schoenlank, 2017; Lynch, 2019). Regarding teacher effectiveness evaluation in EFL/ESL higher education contexts, notable evaluation instrument is based on the learners’ perception of their teachers in the aspect of English proficiency, pedagogical knowledge and socio-affective factors (Park and Lee, 2006). Another perceptive study involves investigating Iranian EFL teachers’ perceptions and attitudes toward teachers’ wants, likes, dislikes, and ideals as to what constitute language teaching (Mazandarani and Troudi, 2021). Given the Stronge evaluation system’s efficacy in helping school administrators to communicate with teachers regarding their instruction in several disciplines (Schoenlank, 2017). It will be interesting to study its effectiveness in evaluating an EFL teacher to promote accountability in EFL education, measure the progress of EFL learners, and the monitoring of EFL teacher quality (Superfine et al., 2012).

In the area of education, many studies on how teachers are represented in movies have been conducted. According to Raimo et al. (2008), teachers in film roles are portrayed as optimistic, sentimental, and unrealistic people (Burbach and Figgins, 1993), sensuality and sexploitation of teens (Hill, 1995; Bauer, 1998), curriculum and instruction paradigm changers (Dalton, 1995; Lasley, 1998). With respect to teachers in different disciplines, movies on physical education (Barbero and Rodriguez, 2017), music (Brand, 2001), English literature (Kurniadi, 2017) teachers have been produced. Moreover, a group of researchers has explored and analyzed teaching strategies adopted by a teacher in the movie *Freedom Writers* (Zulfian et al., 2018). The key objective underlying these studies is to serve two functions. Firstly, they are used in teacher training for reflective practice (Nugent and Shaunessy, 2003). This is because movie offers surrogate experience upon which to develop an educational philosophy since most preservice educational programs do not have sufficient teaching experience to reflect (Ryan and Townsend, 2010). Secondly, they are a valuable tool to foster learning in a classroom due to their audiovisual nature that stimulates emotions (Blasco et al., 2015). This is due to the affordance of moving images in films through a story or plot that provides a complete communicative situation. Such affordance stimulates interest and motivation vital for successful learning (Guest, 1997). In sum, there seems to be a reciprocal relationship between education and school movies.

Mind Your Language is a British sitcom created by Vince Powell and produced and directed by Stuart Allen. The TV show, which was aired between 1977 and 1979, has three seasons. The sitcom plot is centered on Jeremy Brown, a British EFL teacher who helps his immigrant students to learn English language and its culture. Given the EFL pedagogy of laughter that the Show and its protagonist offers to its global audience (Ellingson, 2018). It can be hypothesized that Mr. Brown’s character in the sitcom can be used to model a highly effective EFL teacher, which can serve as a reflective instrument for students in preservice EFL education. Thus, this study evaluates the effectiveness level of Mr. Brown as an EFL teacher in the Mind Your Language TV show by using the Stronge teacher evaluation system. The remaining part of the paper begins with the design of the theoretical framework, followed by reviewing the literature, methodology, results, and discussion and finally, the conclusion, which gives a summary and critiques of the findings. This study provides an exciting opportunity to investigate the personalities and instructional practices of a typical EFL teacher. This is critical to understanding how a teacher should deal with the learners and meet their teaching expectations (Alzeebaree and Hasan, 2020). The identified personalities can also be used as a reflective tool in preservice EFL education and promote instructional accountability among EFL teachers in public schools.

### Theoretical Framework

A character is an individual or other being in a story (such as a novel, play, television series, film, or video game) (DiBattista, 2011). Characters should be plausible and consistent (Faisal, 2011). Alternatively, characterization is the method and process of creating and developing character in fiction (Latif, 2016). Jones (1968, p. 84) notes that characterization is the depiction of an individual’s explicit images. Thus, the true picture of the characters involved in the story is portrayed through their actions, physical appearance, social status, social relationship, and personality of the main characters.

A representation is a written, audio, or visual portrayal of something or an individual (Beltrán, 2018). This term also refers to what picture and written-texts represent, the interpretation that they conceivably express, and how they come to adopt those meanings. It is pointed out that the study of representation also includes a long tradition of critique of how various social groups and identities have been represented in the popular culture more broadly. The author listed some scholarship such as feminist studies and critical race studies, which approach representation with a primary focus on gender, race, ethnicity, sexual orientation, colonization, and its aftermath, class, and ability. Finally, the author stated that one of the critical approaches to studying representation is image analysis. An approach to learning representation that examines media images is one of this study’s primary research methods.

In a summary of Hall (2012), readers and audiences perceive media content as realistic, judge it to be like real life in some meaningful way or respond to it as though it were real. These perceptive responses may depend on the content’s characteristics, such as its theme and genre, which are not fully determined. The author highlights various popular forms of perceived realism. This includes factual realism (whether what is portrayed happened), social realism (whether what is shown is like what one would expect to find in the real world), and narrative realism or narrative coherence (whether the events within a story are well explained and consistent). An empirical study has investigated the social effects of realism on TV messages (Taylor, 2005). The study reveals that the effects on viewers are more important if the message content is perceived to be real rather than fictional (Pouliot and Cowen, 2007). A study has also shown that perceived factuality is likely to influence viewers’ involvement with an audiovisual stimulus, which causes the high intensity of emotional responses (Murry and Dacin, 1996).

In order to evaluate Mr. Brown as an effective EFL teacher, it is essential to analyze his character using the method of characterization. Variables such as his physical appearance, social status, personality, and relationship with students will be considered for the analysis. The researcher will capitalize on the fact that there is yet to be a study on how EFL teacher is represented in popular culture. Thus, based on representation theory, the writer will explore how image media depicts an EFL teacher’s professional and personal characteristics. Lastly, considering the strong connection between reflection and emotion, the researcher will justify the adoption of film for reflective practice. This is based on the notion that factual realism with an audiovisual dimension of perceived realism is potent enough to stir up emotions needed for reflection among preservice EFL teachers. Finally, the successful evaluation of the main character using the Stronge teacher evaluation rubric will demonstrate the role of social realism in the Show. The Show reflects issues that are germane to themes in the real world of EFL education.

**FIGURE 1:**
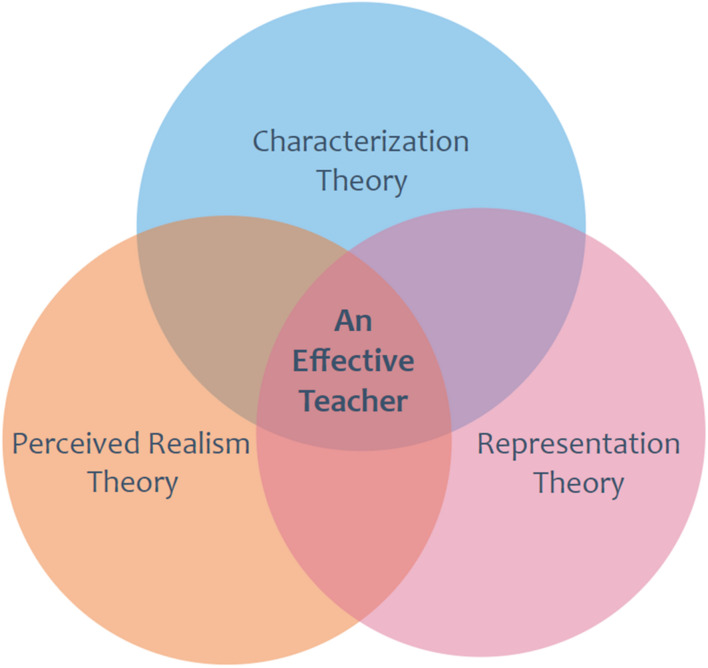
Theoretical conceptualization of an effective teacher in popular culture.

Thus, as shown in Figure 1, my research will be intersecting an effective EFL teacher within the theoretical framework of representation, perceived realism, and characterization. It is where these theories overlap my specific research focus is positioned as demonstrated in the following Venn diagram:

### Literature Review

A large and growing body of literature has investigated good teacher’s attributes and practices. Some view good teachers as professionals with constructivist principles and are committed to facilitating deep, engaged, experientially based, empowering, reflective, and lifelong learning among their students (Duarte, 2013). This view is supported by Harding and Parsons (2011), who writes that a teacher’s pedagogical knowledge depends on his or her ability to engage learners, mediate and negotiate students to participate in their learning actively. Views on the competency and personality of good teachers have been expressed by seeking the opinions of fellow teachers. In a study conducted among early career teachers in Canada, good teachers are described in terms of their core competence (classroom management, adaptive teaching practice, and knowledge of teaching resources) and attribute (optimistic, contributing to the school community, and passion for teaching) (Bernard, 2015). Similarly, a study conducted through an observational analysis concluded that an effective teacher should possess instructional delivery skills, student assessment skills, classroom management skills, and pleasing personalities (Stronge et al., 2011). With regard to the characteristics of EFL Teachers, Park and Lee (2006) compared the opinions of high school English teachers and their Korean students. The study showed that while teachers emphasized the importance of English proficiency, students prioritized pedagogical knowledge. Conversely, Alzeebaree and Hasan (2020) reported that Kurdish high school students placed more importance in English proficiency. Practices such as reading English well, managing classroom properly and being confident and having self-control are also regarded as the quality of effective EFL teachers. It can be argued that there is a copious of studies on the characteristics of an effective teacher. The gap in the literature is the degree of effectiveness of an EFL teacher.

The relevance of teacher performance evaluation is centered on its capacity to foster educational reforms (Stronge, 2018) and as a tool to improve instructional strategies (Goldrick, 2002). For example, it can be used to ascertain the training needs of a teacher by identifying the strengths and inadequacies to facilitate further professional development (Santiago and Benavides, 2009). According to the authors, it can also serve as a tool to make teachers accountable for their performance and relates the level of performance to different actions taking during their career. The past decades have seen publications on the historical evolvement of a teacher evaluation system. For example, the Hay McBer model of teacher effectiveness (McBer, 2000), which introduces “professional characteristics,” “teaching skills,” and “classroom climate” as measures of teacher effectiveness (p. 6), Campbell et al.’s (2003) conception of differentiated model, discussing five domains of difference between their proposed differential model and those of others, and Cheng and Tsui’s (1999) multimodels of teacher effectiveness are a combination of seven models of teacher effectiveness. The most popular one is the Stronge Teacher Effectiveness Performance evaluation standard (Stronge, 2012). It is a comprehensive uniform evaluation system that involves the teacher evaluator (principal or supervisor), and leaders. Its popularity in over 20 countries is based on its uniform evaluation for teachers, principals, and leaders, its comprehensiveness and easy implementation of its seven research-based professional standards custom design for teachers, result-based standards for assessing students’ progress and promoting instructional accountability (Stronge and Tonneson, 2012). Lastly, a study has focused on teachers’ attitudes toward an evaluation system (Paufler and Sloat, 2020). A similar study in the Iranian EFL context revealed that items such as transparency, fairness, multiple measurement, formative evaluation, and cognizance of unequal power relations are critical for an effective evaluation system (Mazandarani and Troudi, 2021). However, far too little attention has been paid to the application of existing teacher evaluation systems in EFL education.

Most current researchers are paying attention to how teachers can acquire character traits critical and essential for successful teaching. The impact of Mindfulness Meditation (MM) on trait personality characteristics is one of the primary studies (Fabbro et al., 2020). MM has been proven to foster positive improvements in teachers’ personality/character traits and self-concept (Campanella et al., 2014; Crescentini and Capurso, 2015). One of the most common instruments employed in this line of study has been the Big Five Personality Inventory BFI) (Costa and McCrae, 1992), addressing five different personality traits: (a) extraversion (related to positive affectivity and sociality); (b) neuroticism (reflecting negative affectivity, such as anxiety or depression); (c) agreeableness (encompassing empathy, cooperation, and altruism); (d) conscientiousness (related to self-discipline, self-efficacy, and control); and (e) openness to experience (reflecting curiosity for cognitive exploration). The instrument has been used to examine preservice teachers’ personality trajectories (Corcoran and O’Flaherty, 2016) and compare the personality traits of preservice elementary school teachers in Spain and Turkey (Perkmen et al., 2018). These established studies have important implications for developing personality traits critical for effective teaching practices in ESL/EFL education.

Teachers’ public perceptions are influenced mainly by popular culture (Dalton, 2010), and especially movies (Resnick, 2018). That explains the copious of studies on constructing teachers’ identity by the media (Farhi, 1999; Kirby, 2016). Teachers are portrayed as heroic (Farhi, 1999) and professionals that persevere during frustration (Edelman, 1990, p. 18). The trajectory of teacher representation in movies has also been argued on how male and female teachers were depicted. In contrast to male teachers who are shown to possess masculine power in a social space, female teachers are portrayed as professional in conflict with maternal and allure powers in a domestic space (Dalton and Linder, 2008). Recent representation is now focusing on the concept of “goodness” or “highly effective teacher” (Dalton, 2013). Some authors focus on the personal attributes of teachers in the movie, like *Ms. Sinclair* (Kurniadi, 2017). In the same vein, Zulfian et al. (2018) depict teaching strategies and class management skill demonstrated by a teacher in a movie like (*Freedom Writer*). In sum, these studies provide important insights into how teachers’ personalities and professional practices are constructed in film.

Reflection is regarded as a vital core practice in preservice teacher education (Robichaux and Guarino, 2012). One effective way to promote reflection in this program is to use films as the platform to stimulate interest, motivation and reflection (Ryan and Townsend, 2010). Various studies have demonstrated the positive role of films in enhancing the professional practices of student teachers. Tan (2006) found that films help students to reflect on their personal beliefs and assumptions, solve their personal and professional obstacles and apply the implications to the local context, then meet their students’ specific educational needs, and finally, review and change personal instructional goals, methods, and resources. A study design to help student–teachers to reflect on their complex and sensitive aspect of teacher identity involves identifying educational taboos in Half Nelson and All Things Fair (Van Beveren et al., 2018). The result shows that the rhetorical perspective adopted as a methodological approach helps students to identify and understand the complex and sensitive aspects that affect teaching and teachers’ identities. In a nutshell, student–teachers can develop their professional identity through admired teachers’ depicted in media images (Kirby, 2016).

Given this backdrop, there is a lacuna in the literature with regard to reporting EFL teacher’s representation in films and the application of teacher performance evaluation system (TEPES) for assessing EFL teacher effectiveness. Thus, having identified Mind Your Language TV show as the only existing sitcom that depicts the personal and professional characteristics of a typical EFL teacher, the questions underlying this research was:

What is the effectiveness level of Mr. Brown (an EFL teacher) in the Mind Your Language TV show using Stronge teacher evaluation system?

## Materials and Methods

### Research Design

Previous studies on movie rely on researcher’s interpretation. The methodology used by the investigator is qualitative descriptive research. In a qualitative research design, the meaning is not explicitly revealed, but it is constructed (Schreier, 2012). The study is intended to decode the signs that cause the main character “*Mr. Brown*” to be a real representation of an effective EFL teacher in the TV show “*Mind Your Language*.” Sandelowski (2000, p. 4) says that qualitative descriptive designs are typically an eclectic but realistic and well-considered mix of sampling, and data collection, evaluation, and representational techniques.

### Source of Data

The data for this analysis was extracted from episodes of *Mind Your Language*. It is a British sitcom that premiered on ITV in 1977. It was produced by London Weekend Television and directed by Stuart Allen. Three series were made by LWT between 1977 and 1979 and briefly revived in 1985 (or 1986 in most ITV regions) with six of the original cast. The Show is set in an adult education college in London and focuses on the class in English as a Foreign Language directed by Mr. Jeremy Brown (Barry Evans), who is the main character. The plot of Mind Your Language focuses on Jeremy Brown, an EFL teacher who works at a school managed by Dolores Courtney, and his experiences that his students to learn English and its culture. It is a show of over 40 episodes with four seasons. The sitcom was chosen as a source of data collection because it provides EFL pedagogy of laughter to its global audience (Ellingson, 2018). That is, the Show as an edutainment adopts humor to teach and motivate viewers across the world to learn English and its culture. This reduces anxiety, tension, and stress (Berk, 2000) critical for successful passive learning of English among the viewers (Garner, 2006).

### Instrumentation

Concerning EFL teacher effectiveness, parameters such as English proficiency, pedagogical knowledge, and socio-affective factors have been adopted (Park and Lee, 2006). However, the instrument is developed based on the perception of learners. It does not take account of fellow teachers and school administrators, nor does it involve teachers’ self-perception. This drawback has led to the creation of Teacher Performance Evaluation System (TPES) by James Stronge (Dinwiddie County Public Schools, 2012). TPES is a rational and rigorous evaluation system that provides sufficient detail and accuracy so that both teachers and evaluators reasonably understand job expectations. It uses a two-tiered approach to define teacher performance expectations consisting of seven criteria and multiple performance metrics. Teachers will be graded based on the performance standards using performance appraisal rubrics. This instrument’s choice to assess the character is based on its robustness in covering all dimensions of teacher quality, its popularity as well as acceptability in over 20 countries (Stronge and Tonneson, 2012). Reference to the dimensions of a past instrument developed by Park and Lee (2006) overlap with some of the performance indicators of Stronge’s instrument. However, the latter’s instrument does not cover EFL teachers’ professionalism, an aspect critical for assessing their professional relationship with other teachers and school and student progress, which serves as a self-assessment skill that helps teacher measure student learning progress. The TPES provides a balance between structure and flexibility (Dinwiddie County Public Schools, 2012). According to the source, it is prescriptive in that it describes common purposes and standards, thereby guiding effective instructional practice. Furthermore, it provides flexibility, thereby allowing for creativity and individual teacher initiative. Stronge et al. (2008) argued that the tool was initially developed to promotes teachers’ professional development and performance accountability as well as schools’ improvement. Also, Dinwiddie County Public Schools (2012) believed that the performance indicators are constructed to illustrate observable, tangible behaviors for each standard. They are the types of performance that will occur if a standard is being fulfilled.

Furthermore, the list of performance indicators is not exhaustive. In other words, it is not supposed to be prescriptive or a checklist. However, based on the number of indicators meet in each performance standard, teachers will be rated in the order of highly effective (teacher surpasses the established standard), effective (teacher meets the expected standard), partially effective (teacher is often performing below the established standard), and ineffective (teacher perform consistently perform below the established standard), respectively. For example, an effective teacher is expected to meet the indicators in each of the seven performance standards. Thus, Stronge Teacher Effectiveness Performance Evaluation System (STEPES) was adapted to evaluate Mr. Brown as an effective teacher. In formulating the Performance Standard Indicators (PSI), and the accompanying evidence for each standard, the discipline (EFL), previous classroom observation form adopted in different contexts (Dinwiddie County Public Schools, 2012) and the age group (adult) were considered. Stronge (2012) checklist is divided into seven performance standards (professional knowledge, instructional planning, instructional delivery, assessment of/for learning, learning environment, professionalism, and student progress). Each performance standard and the corresponding indicators are adapted and redefined as follows:

**Professional knowledge:** The teacher demonstrates an appreciation of the curriculum, subject matter, and the formative needs of students by providing relevant learning experiences.
**Instructional planning:** The teacher plans to use the school’s curriculum, appropriate methods, tools, and data to meet the needs of all students.
**Instructional delivery:** The teacher promotes learning by utilizing an assortment of instructional techniques to meet students’ needs.
**Assessment of and for student learning:** The instructor systematically utilizes all relevant information to assess student academic progress, guide instructional content, and delivery methods and provide students with timely feedback.
**Learning environment:** The instructor makes use of tools, routines and procedures to provide an atmosphere that is respectful, constructive, secure, student-centered, and conducive to learning.
**Professionalism:** The teacher is committed to professional integrity, communicates efficiently, takes responsibility for professional development and participates in it, and resulting in better students’ learning outcome.
**Student progress:** The teacher’s job results in acceptable, and measurable student academic progress. That is, teacher’s mandate to foster students’ learning is fulfilled.

### Data Collection Procedure and Justification

The procedures of data collection used are visual observation and documentation. In analyzing the data, the descriptive analysis technique is used to identify and analyze the teaching strategies and the teacher’s personality in the Show. Among the four seasons aired during the chosen period, three were selected for analysis. This is because the fourth seasons’ unavailability on YouTube (the most extensive video sharing and depository in the world). The YouTube playlist of the Show uploaded by Daily Laughter, Asian Streysart, and other individuals were chosen interchangeably for analysis in missing episodes. Season 1, 2, 3 have 13, 8, and 8 episodes, respectively. Suppose one of the episodes selected as part of a multiple-part episode, all episodes from the series were considered and analyzed. The number of selected episodes was based on a study by Manganello et al. (2008) that sampling seven episodes from a TV season was necessary to draw character-based conclusions. This research represents a much more robust and systematic approach than this recommendation, similar to Lasekan (2021) method.

Mind Your Language was chosen because it is the only existing TV show that depicts an EFL teacher’s character. The TV Series’ choice over a movie is based on the fact that the latter offer more elaborate content for analysis, being a 3-year TV show with over 40 episodes. The investigator viewed each meaningful contact exchange multiple times. First the researcher watched the entire episode, taking notes on which interaction met the evidential characteristic and teaching techniques formulated in the checklist of Stronge Teacher Effectiveness Performance Evaluation. The researcher transcribed the correspondence between the instructor and a student or students in the second viewing. The researcher took notes on the interaction meaning in the final viewing, subtle verbal intonations, and any non-verbal images. Next, each of the main interactions was coded and corresponded to the seven performance standards. Professional knowledge, instructional planning, instructional delivery, assessment of/for learning, learning environment, and professionalism and student progress.

### Data Analysis

In this study, the qualitative content analysis research method was used, and the data analysis is conducted according to the adapted procedures put forward by Lacko (2011). The first step in content analysis is the determination of research objectives. In this study, the researcher’s main objective was to portray Mr. Brown’s character in Mind Your Language TV show as an effective teacher using STEPES as a checklist for evaluation. The researcher aimed to identify the teaching strategies and characteristics of the main character that match the seven performance standards of the evaluation system.

The essential unit of the investigation was analyzing of the interaction between the teacher and students. The interaction had to have lasted at least 5 s to count as important and is expected to be crucial for advancing the story. Significance assessments are based on the rationale of Ye and Ward (2010) analysis unit.

Manifest and latent content analysis were the data analysis used in this report. They are described in details as follows:

Manifest Content Analysis: It refers to the process of encoding visible or surface content (Neuman, 2000). In other words, quantitative content analysis entails categorizing the material according to predefined and precise characteristics (Dowler, 2004).

Latent Content Analysis: It involves the process of determining the underlying, implicit meaning of a text’s content (Neuman, 2000). Thus, the meaning of words, phrases, or terminology is subjective, necessitating some semantic analysis (Dowler, 2004).

Using a single coder for this study is justified and reliable because the coder is knowledgeable about the subject matter (Campbell et al., 2013). The researcher’s knowledge is based on years of watching every episode of the series. Similar to study conducted by Lasekan (2021), several steps were taken as a lone researcher to improve the analysis’s reliability. This includes discussing the coding and analysis with other colleagues who are strong followers of the show. This process which is called “member checking” entails sharing coded field note excerpts and discuss coding and analysis helps in reconciling views on the rating of professional and personal characteristics depict by Mr. Brown (Burant et al., 2007).

In this study, with the help of the Stronge Teacher Effectiveness Performance checklist (Stronge, 2012), explicit categories were developed for the analysis of Show. This involves interpreting and matching with adapted Stronge’s proposed evidential traits and strategies of each performance standard with personal characteristics of the teacher and its teaching strategies in both in-class and out-class. This is followed by coding of manifest and latent themes. The researcher matches the themes with the evidential characters and teaching strategies of each performance standard.

To determine the degree of effectiveness of Mr. Brown as an EFL teacher, a seven-point Likert scale ranging from ineffective (1), lowly effective (2), slightly effective (3), neutral effective (4), moderately effective (5), very effective (6), and highly effective (7) (Joshi et al., 2015). The scale is used to correspond to the TEPES 7 performance standards. Therefore, the total number of personal or professional characteristics of Mr. Brown evidenced in all the performance standards determines his effectiveness as a teacher.

## Results

This study identifies and evaluate the personal and professional characteristics of Mr. Brown in the “Mind Your Language” TV show using Stronge teacher evaluation system. As shown in Supplementary Table 1, evidence from different observation forms of each performance standard and the corresponding indicator revealed that Mr. Brown is a highly effective EFL teacher.

Professional knowledge involves teachers showing an understanding of the subject and meeting the need of the students. Mr. Brown knowledge of English as a native speaker of the language and its qualification as an EFL teacher is expressed in Season 1, Episode 1. The school principal claimed that she requested a female teacher, but the Ministry of Education sent her a male teacher. Thus, for working with the Ministry of Education, it can be argued that Mr. Brown is qualified and licensed EFL teacher. Regarding the establishment of English communication to ensure that students needs are met as adult learners, Mr. Brown adopts the concept of repetition by checking students’ understanding. He is also aware that the students are immigrants in Britain; thus, they should learn and practice tasks that helps them to communicate easily in British society. For example, he gave them a task that is suitable for an adult. They were asked to do sightseeing tour in England to practice their English conversation.

Instructional planning is the second performance standard. The indicator includes a paced lesson, creating a lesson that causes critical thinking and adapting the curriculum to meet the students’ needs. With the way the teacher instructs, it suggests that the teacher plans his lesson before the class begins. For example, the Crossroad Game for class activity was prepared on the blackboard before the beginning of the class. In Season 2, Episode 2, Sentence Structure development was taught systematically. That is an indication that the lesson was systematically planned. Also, the teacher adopts the standard curriculum to meet student needs. This is manifested in Season 1, Episode 2 when he justified his personalized curriculum to the Ministry of Education’s local inspector.

Instructional delivery centered mainly on the strategies adopted by Mr. Brown. This includes using questioning techniques on a wide variety of topics. Topics such as music, the royal family of England and Shakespeare were adopted to teach British culture. This helps in facilitating a higher level of thinking. The teacher helps the students in providing feedback to each other. Being a multicultural class, the students collaborate by helping each other interpret and explain the teacher’s idea in the classroom. In addition, numerous instructional strategies such as the art of conversation, joke, game, debate, and excursion were adopted to facilitate learning inside and outside the classroom.

Assessment of/for learning focus on a systematic way of providing feedback to students and measuring students’ progress. One of the scenes shows how the teacher gives feedback to students based on the previously assigned homework. Students were also informed about the structure of their lower Cambridge examination. With respect to preparing students for the final exam, a mock is given as a formative test to prepare students for the final exam.

Learning environment involves creating a conducive environment for effective learning. Being a classroom full of students from different countries, maintaining an inclusive environment to make the student comfortable in the classroom is critical. Arranging the class in a manner to facilitate effective learning is also demonstrated. For example, the classroom is rearranged by asking two students to sit in front of the class while using the art of conversation to facilitate speaking practice. The teacher respects his Japanese student by greeting him Japanese to promote cultural acceptance. An inclusive classroom is shown when extra attention is given to new students with lower level of English proficiency. Several occasions revealed how conflict is resolved between students from different cultures and religions. Especially between a Sikh and Muslim student. An atmosphere where students provide one another moral support for learning through encouragement was revealed in the Show.

Professionalism as one of the performing standards of a good teacher involves teachers upholding and committing to professional learning. The main character does not demonstrate this practice. Still, professional ethics concerning manner and dressing are revealed in the Show. The teacher is seen dressing professionally throughout the Show. An ethical relationship is also maintained with all of his students, especially toward a student expressing an unsolicited flirtatious attitude toward him. Several instances are also shown when teacher communicate with the family member of his student to resolve their personal problems. Thus, he can be described as a caring teacher. Furthermore, a professional and collegiate relationship is built between the teacher and principal to improve student learning. For example, the principal and teacher work together on how to improve students’ learning outcomes.

Student progress involves assessing the work of the teacher result in an acceptable and appropriate student academic progress. The Show demonstrates evidence in the improvement of one of the student’s oral communication named Jamila. The student was not fluent in the first season of the Show, but she becomes fluent by the end of the second season. The teacher is also monitored continuously by the principal to ensure that the goal of fostering the students’ English proficiency is fulfilled.

## Discussion

It is interesting to know that the main character is a highly effective modern EFL teacher. This is because Mr. Brown fulfilled the standard performance indicator of each of the seven performance standards. This includes professional knowledge, instructional planning, instructional delivery, assessment of/for learning, learning environment, professionalism, and student progress. That implies Mr. Brown as an EFL teacher has sustained high performance over period of time, his behaviors have strong positive impact on learners and school climate and can serve as a role model to other EFL teachers (Stronge, 2018). In other words, based on the set standard to assess Mr. Brown’s effectiveness as EFL teacher, the character depicts all the PSI except in the area of pursuant of professional development and following the local curriculum in the Show. The inadequacy of the latter was justified through the need to use personalized curriculum to meet the students’ specific needs. In sum, the effectiveness level of Mr. Brown as an EFL teacher, which is established through the analysis of his character provides a clear understanding of how the profession is represented in a popular culture. Theoretically, the researcher has succeeded in depicting the character (DiBattista, 2011) of Mr. Brown as an EFL teacher through its interaction with the students in the Show. As a result of this, the representation (Beltrán, 2018) of an EFL teacher can be understood in the TV show. The teacher’s positive assessment with the use of Stronge TEPES is a demonstration of perceived reality (Hall, 2012), which is capable of stirring up the emotion needed for reflective practice among preservice teachers. This study is consistent with previous research on the role of film in shaping the belief system of people (Mariani, n.d.).

In consistence with the study conducted by Zulfian et al. (2018), some of the teaching strategies adopted by Mr. Brown in this work are similar to the ones identified in the *Freedom Writers* movie. This included games, field trips, and class discussion. However, in contrast to previous findings (Brand, 2001; Barbero and Rodriguez, 2017), this is the first study to evaluate the effectiveness of an EFL teacher by considering a TV Series that avail the considerable amount of content for identifying the characteristics of the teacher. In addition, unlike the previous study that focuses on a teacher’s personality (Kirby, 2016), this study adopts a robust and well-acceptable approach to evaluate the effectiveness of a teacher by considering both the personalities and other significant criteria that define a 21st-century teacher. Moreover, the TEPES as an instrument is validated in EFL education because some of its performance standards is similar to previous tool developed by Park and Lee (2006) to assess the characteristics of EFL teachers. The overlap items in both instruments are encapsulated in the subject matter knowledge, pedagogical knowledge, and socio-affective skills identified by the authors, and it has been used to assess the perception of Kurdish high school students toward the characteristics of their EFL teachers successfully (Alzeebaree and Hasan, 2020). Therefore, adopting TEPES to evaluate EFL teachers is effective in identifying the key competencies of an EFL teacher.

Having established Mr. Brown as a highly effective EFL teacher, it can be argued that the Show is a valuable popular culture that perpetuates the personalities, teaching strategies, classroom management, and professionalism of an English teacher. As a highly effective EFL teacher, Mr. Brown can serve as a role model to other EFL teachers. That is, his personalities can be used to promote reflective practice among preservice English teachers through journal writing, which is an efficient way to boost reflections after the students have watched the visual (Holly, 2002). The character of Mr. Brown can be used to carry out self-examination of one’s aims, beliefs, assumptions, and actions (Pollard and Tann, 1987), resolve one’s own personal and professional obstacles (Dewey, 1933), understand the socio-cultural implications of teaching and learning (Zeichner and Liston, 1987) and modify one’s skills in response to the learners’ needs (Darling-Hammond, 2000). Such reflections will empower a preservice teacher to develop their intellect and learn autonomously to unravel the effective approach to teaching EFL learners. Furthermore, this Show can trigger reflection regarding the education myth (Van Beveren et al., 2018). For example, a debate on teachers’ gender staminality to work in a harsh condition was mentioned in the Show. The principal’s initial belief is that Mr. Brown will not last in his teaching job because he is a man. However, the Show proves otherwise by portraying a male’s capability to teach EFL effectively in a chaotic and multicultural classroom. Finally, the character of Mr. Brown is suitable enough to teach and promote teacher effectiveness in preservice and in-service EFL education. Other than the adapted teacher evaluation system of Stronge, the researcher can argue that if the students were to evaluate Mr. Brown, they would have rated him as a good teacher. Their rating would have been based more on his personality rather than his pedagogical skill or qualification to teach English. For example, In Season 3, Episode 4, the students fought for Mr. Brown’s reinstatement when he temporarily lost his job while he was trying to secure a better-paid job in another school. According to one of the students, she testifies, “a new teacher won’t be as nice as Mr. Brown.” With respect to the local inspector official from the Ministry of Education, Mr. Brown is commended as a remarkable man for his teaching style. The official says in Season 1, Episode 2, “His teaching methods may be revolutionary, but they appear to be working.” Another profound practice shown by the main character is assigning homework as a positive reinforcement to improve learning outcomes. The writer observes that students are interested in English, but they are easily distracted during classroom activities. Thus, whenever students are not making significant progress in their learning or causing a distraction during class activities, the teacher assigns more homework to his students as a form of positive reinforcement. Another important practice is the taking of attendance at the beginning of the class. It can be argued that the teacher understands the importance of attendance so that the learning outcome can be expedited. Finally, it is essential to reveal the flaws of the teachers. These flaws are related to his professional ethics. In Season 1, Episode 2, the teacher was seen coming late to the class. Also, while the principal was testing the students’ progress of learning in the teacher’s presence, Mr. Brown was trying to provide answers to the students so that they could answer the principal’s question. Thus, even though he can be considered a quality teacher by an acceptable and standardize teacher evaluation system, the teacher still have flaws that can trigger reflection among preservice teachers, which helps them change or modify their values, beliefs, and actions (Tan, 2006).

As mentioned in the literature, studies have identified an effective EFL teacher’s characteristics based on perceptive views of EFL learners in different contexts (Mazandarani and Troudi, 2021). The identified personality traits portrayed by Mr. Brown are consistent and overlap with those of Park and Lee (2006), who identified pedagogical knowledge, English proficiency and socio-affective skills as characteristics of effective teachers. These findings may help EFL teacher to understand how they can be dealing with the learners and also meet their teaching expectations (Alzeebaree and Hasan, 2020).

The identified personal attributes in this study have offered an opportunity to investigate the development of personality traits among EFL teachers. Given a considerable amount of literature that has been published on the role of MM in boosting teachers’ personalities (Crescentini and Capurso, 2015; Fabbro et al., 2020), little is known about its application in ESL/EFL education. Thus, a further study focusing on operationalizing MM for the formation of identified personal and professional traits among EFL teachers is suggested.

One of the critical factors responsible for the popularity of implementing the Stronge TEPES (2006) is the increasing demand for instructional accountability among teachers in several contexts (Lynch, 2019). According to the author, the demands speak to higher instructional expectations and the need for the school administrator to be abreast of instructional practices occurring in the classroom more diligently. In its application, numerous school administrators believe the evaluation system is valuable in communicating with teachers regarding their instruction (Schoenlank, 2017). As far as this study is concerned, TEPES has been proven to be efficient in evaluating the effectiveness of an EFL teacher. That suggests that the system can catalyze English class improvement, professional growth, and performance accountability of teachers (Stronge et al., 2008). Moreover, this instrument can help school administrators to set higher expectations for teaching and learning English at public and private schools. They will help English teachers carry out purposeful discussions that will yield good results in teaching and learning English (Zarei et al., 2019). In addition, the evaluation system can be used to foster reforms (Stronge, 2018) and enhance instructional strategies in ESL/EFL education (Goldrick, 2002). Moreover, it can serve as a tool to carry out the Strengths, Weaknesses, Opportunities, and Threats (SWOT) analysis of EFL teachers to facilitate their professional development and make teachers accountable for their performance (Santiago and Benavides, 2009).

The sitcom and its protagonist were chosen as sources of data collection because it offers EFL pedagogy of laughter to its global audience (Ellingson, 2018). The current study has shed more light on how Mr. Brown uses jokes and humor to carry out his English instruction and management of the classroom. That speaks to the importance of using appropriate humor to facilitate foreign language learning in the classroom (Jansson, 2016). Also, Mr. Brown exceptional skill in using a game to facilitate language learning confirmed the pertinence of his teaching practice in the 21st-century classroom. This practice further supports the importance of ludic pedagogy, a teaching philosophy that emphasizes the importance of play, games, and fun while maintaining academic rigor (Broussard, 2011). Lastly, the relevance of this Show in today’s modern classroom is demonstrated in the way a modern teacher evaluation system rated the quality of an EFL teacher that lives in the 1970s highly. This is an indication that as long as education is always in a state of transition, there will always be places for old teaching approaches within the EFL education (Luther, 2000).

## Conclusion

The current study’s main goal was to use the Stronge teacher evaluation system to assess the effectiveness level of Mr. Brown as an EFL teacher in Mind Your Language TV show. This study has shown that the character is a highly effective teacher because his personal and professional characteristics are evidenced by all the seven performance standards of the evaluation system. The results of this study indicate that his character in the Show as a quality EFL teacher can be used as a positive role model and reflective tool to train preservice EFL teachers on how to enhance their professional identities and instructional practices. Consequently, it helps them to become highly effective teacher. Concerning EFL teachers in public school, the personal characteristics identified in this study can be used by school administrators to promote instructional accountability among their EFL teachers. This research add to a growing body of literature on teachers’ representation in movies and its implication in preservice education. This is the first study reporting on the representation of EFL teachers in a TV show. In addition, this is the first time that the Stronge teacher evaluation system has been used successfully to assess a teacher’s image in popular culture. Thus, this assessment tool can be used to evaluate teachers’ personal and professional characteristics in different school movies. Finally, the generalizability of these results is subject to certain limitations. For instance, Mr. Brown’s effectiveness is based on his character as an EFL teacher that teaches only adult learners. Also, the dataset was analyzed by a single rater; therefore, the analyses were based on the researcher’s interpretation. Thus, his effectiveness should be interpreted with caution when it comes to teaching both young and adult EFL learners. Another limitation lies in the fact that only a qualitative method was adopted. Thus, triangulation method is required for collecting more robust data. More research is needed to better understand how this TV show can be used to promote philosophical reflections in preservice English teachers’ education.

## Data Availability Statement

The raw data supporting the conclusions of this article will be made available by the authors, without undue reservation.

## Author Contributions

The author designed the research and wrote the manuscript.

## Conflict of Interest

The author declares that the research was conducted in the absence of any commercial or financial relationships that could be construed as a potential conflict of interest.

## Publisher’s Note

All claims expressed in this article are solely those of the authors and do not necessarily represent those of their affiliated organizations, or those of the publisher, the editors and the reviewers. Any product that may be evaluated in this article, or claim that may be made by its manufacturer, is not guaranteed or endorsed by the publisher.

## Supplementary Material

Supplementary Table 1 Assessment of Mr. Brown effectiveness as an EFL teacher using Stronge teacher evaluation system.

## References

[B1] Alrefaee Y. Al-Ghamdi N. (2019). Refusals among Yemeni EFL learners: a study of negative pragmatic transfer and its relation to proficiency. *Asian EFL J.* 25 191–214.

[B2] Alzeebaree Y. Hasan I. A. (2020). What makes an effective EFL teacher: high school students’ Perceptions. *Asian ESP J.* 16 169–183.

[B3] Ansari U. Malik S. K. (2013). Image of an effective teacher in 21st century classroom. *J. Educ. Instr. Stud. World* 3 61–68.

[B4] Barbero J. I. G. Rodríguez H. C. (2017). Physical education teachers (re)presentation in the movies. *Cultura Ciencia Y Deporte* 12 161–171. 10.12800/ccd.v12i36.945

[B5] Bauer D. M. (1998). Indecent proposals: teachers in the movies. *Coll. English* 60 301–317. 10.2307/378559

[B6] Beltrán M. (2018). *Representation. In The Craft of Criticism.* Milton Park: Routledge, 97–108.

[B7] Berk R. A. (2000). Does humor in course tests reduce anxiety and improve performance? *Coll. Teach.* 48 151–158. 10.1080/87567550009595834

[B8] Bernard Jr, M. (2015). *The Good Teacher**: An Investigation of the Core-Competencies and Attributes of an Effective Educator.* Available online at: https://tspace.library.utoronto.ca/bitstream/1807/68699/1/Bernard_Martin_P_201506_MT_MTRP.pdf (accessed September 9, 2021).

[B9] Blasco P. G. Moreto G. Blasco M. G. Levites M. R. Janaudis M. A. (2015). Education through movies: improving teaching skills and fostering reflection among students and teachers. *J. Learn. Arts* 11:n1.

[B10] Brand M. (2001). Reel music teachers: use of popular films in music teacher education. *Int. J. Music Educ.* 38 5–12. 10.1177/025576140103800102

[B11] Broussard J. (2011). *Playing Class: A Case Study Of Ludic Pedagogy.* Baton Rouge, LA: Louisiana State University.

[B12] Burant T. J. Gray C. Ndaw E. McKinney-Keys V. Allen G. (2007). The rhythms of a teacher research group. *Multicult. Perspect.* 9 10–18. 10.1080/15210960701333674

[B13] Burbach H. J. Figgins M. A. (1993). A thematic profile of the images of teachers in film. *Teach. Educ. Q.* 20 65–75.

[B14] Campanella F. Crescentini C. Urgesi C. Fabbro F. (2014). Mindfulness-oriented meditation improve self-related characters scales in healthy individuals. *Compr. Psychiatry* 22 1269–1278. 10.1016/j.comppsych.2014.03.009 24746260

[B15] Campbell J. L. Quincy C. Osserman J. Pedersen O. K. (2013). Coding in-depth semistructured interviews: problems of unitization and intercoder reliability and agreement. *Sociol. Methods Res.* 42 294–320. 10.1177/0049124113500475

[B16] Campbell J. Kyriakides L. Muijs D. Robinson W. (2003). Differential teacher effectiveness: towards a model for research and teacher appraisal. *Oxf. Rev. Educ.* 29 347–362. 10.1080/03054980307440

[B17] Cheng Y. C. Tsui K. T. (1999). Multimodels of teacher effectiveness: implications for research. *J. Educ. Res*. 92 141–150. 10.1080/00220679909597589

[B18] Colker L. J. (2008). Twelve characteristics of effective early childhood teachers. *YC Young Child.* 63:68.

[B19] Corcoran R. P. O’Flaherty J. (2016). Personality development during teacher preparation. *Front. Psychol.* 7:1677. 10.3389/fpsyg.2016.01677 27877143 PMC5099232

[B20] Costa P. T. McCrae R. R. (1992). *Revised NEO Personality Inventory (NEOPI–R) and NEO Five-Factor Inventory (NEO-FFI): Professional Manual*. Odessa, FL: Psychological Assessment Resources.

[B21] Crescentini C. Capurso V. (2015). Mindfulness meditation and explicit and implicit indicators of personality and self-concept changes. *Front. Psychol. Psychol.* 6:44. 10.3389/fpsyg.2015.00044 25688222 PMC4310269

[B22] Dalton M. M. (1995). The Hollywood Curriculum: who is the ‘good’teacher? *Curric. Stud.* 3 23–44. 10.1080/0965975950030102

[B23] Dalton M. M. (2010). *The Hollywood Curriculum: Teachers in the Movies*, Vol. 256. Bern: Peter Lang.

[B24] Dalton M. M. (2013). Bad Teacher is bad for teachers. *J. Pop. Film Telev.* 41 78–87.

[B25] Dalton M. M. Linder L. R. (2008). *Teacher TV: Sixty Years Of Teachers On Television*, Vol. 320. Bern: Peter Lang.

[B26] Darling-Hammond L. (2000). Teacher quality and student achievement. *Educ. Policy Anal. Arch.* 8:1. 10.14507/epaa.v8n1.2000

[B27] Dewey J. (1933). *How We Think: A Restatement of the Relation of Reflective Thinking to the Educative Process*, Vol. 8. Boston, MA: D.C. Heath & Co Publishers.

[B28] DiBattista M. (2011). *Novel characters: A genealogy.* Hoboken, NJ: John Wiley & Sons.

[B29] Dinwiddie County Public Schools (2012). *Teacher Performance Evaluation System.* Available online at: *https://www.dinwiddie.k12.va.us/app/uploads/sites/2/2019/02/TeacherPerformanceEvaluationSystemAugust2012.pdf* (accessed September 9, 2021).

[B30] Dowler K. (2004). Comparing american and canadian local television crime stories: a content analysis. *Can. J. Criminol. Criminal Justice* 46 573–596. 10.3138/cjccj.46.5.573

[B31] Duarte F. P. (2013). Conceptions of good teaching by good teachers: case studies from an Australian university. *J. Univ Teach. Learn. Pract.* 10:5.

[B32] Edelman R. (1990). *Teachers in the movies, American Educator:The Professional. Journal of the American Federation of Teachers* 7. New Jersey, NJ: ERIC - Institute of Education Sciences.

[B33] Ellett C. D. Teddlie C. (2003). teacher evaluation, teacher effectiveness and school effectiveness: perspectives from the USA. *J. Pers. Eval Educ.* 17 101–128. 10.4324/9780203020234-12

[B34] Ellingson L. L. (2018). “Pedagogy of laughter: using humor to make teaching and learning more fun and effective,” in *Teaching with Sociological Imagination in Higher and Further Education*, eds Matthews C. R. Edgington U. Channon A. (New York, NY: Springer), 123–134. 10.1007/978-981-10-6725-9_8

[B35] Fabbro A. Fabbro F. Capurso V. D’Antoni F. Crescentini C. (2020). Effects of mindfulness training on school teachers’ self-reported personality traits as well as stress and burnout levels. *Percept. Mot. Skills* 127 515–532. 10.1177/0031512520908708 32122249

[B36] Faisal (2011). *Analysis of Main Character in Bruce Almighty Movie Viewed from Personality Traits Theory by Costa and McCrae.* Banten: Islamic State University of Syarif Hidayatullah.

[B37] Farhi A. (1999). Hollywood goes to school recognizing the superteacher myth in film. *Clearing House* 72 157–159. 10.1080/00098659909599618

[B38] Garner R. L. (2006). Humor in pedagogy: how ha-ha can lead to aha! *Coll. Teach.* 54 177–180. 10.3200/ctch.54.1.177-180

[B39] Goldrick L. (2002). *Improving Teacher Evaluation To Improve Teaching Quality. Issue Brief.* Available online at: https://files.eric.ed.gov/fulltext/ED480159.pdf (accessed September 9, 2021).

[B40] Goodwin A. L. Kosnik C. (2013). Quality teacher educators= quality teachers? Conceptualizing essential domains of knowledge for those who teach teachers. *Teach. Dev.* 17 334–346. 10.1080/13664530.2013.813766

[B41] Gordon L. M. (2012). *Good Teaching Matters, Teachers Matter, and Teacher Education Matters. Online Submission.* Available online at: https://files.eric.ed.gov/fulltext/ED538614.pdf (accessed September 9, 2021).

[B42] Gower G. Byrne M. (2012). “Becoming a culturally competent teacher: Beginning the journey” in. *Reform And Resistance In Aboriginal Education* Vol. 379. eds Beresford Q. Partington G. Gower G. Crawley, WA: UWA Publishing.

[B43] Grant L. W. Stronge J. H. Xu X. (2013). A cross-cultural comparative study of teacher effectiveness: analyses of award-winning teachers in the United States and China. *Educ. Assess. Eval. Account.* 25 251–276. 10.1007/s11092-013-9170-1

[B44] Guest M. (1997). “Film dynamics in the english language classroom,” in *Proceedings of the Sixth International Symposium On English Teaching*, Taipei, 171–182.

[B45] Hall A. (2012). *Perceived Realism.* Oxford: Oxford University Press.

[B46] Harding K. Parsons J. (2011). Improving teacher education programs. *Aust. J. Teach. Educ.* 36:4. 10.14221/ajte.2011v36n11.7

[B47] Hill D. (1995). Tinseltown teachers. *Teach. Mag.* 6:4045.

[B48] Holly M. L. (2002). *Keeping a Professional Journal*. Sydney: UNSW Press.

[B49] Jansson D. (2016). *Humor as Pedagogy: A Geographical Perspective*. Uppsala: Uppsala University, 45–52.

[B50] Jones E. H. (ed.) (1968). *Outlines of Literature: Short Stories, Novels, and Poems.* Basingstoke: Macmillan.

[B51] Joshi A. Kale S. Chandel S. Pal D. K. (2015). Likert scale: explored and explained. *Curr. J. Appl. Sci. Technol.* 7 396–403. 10.9734/BJAST/2015/14975

[B52] Kirby D. C. (2016). *The Influence Of Teacher Media Images On Professional Teacher Identities* Doctoral dissertation, UCL, University College London.

[B53] Kurniadi K. (2017). *Representation of the Effective Teacher of the Main Character (Ms. Sinclair) in Film “The English Teacher”*, Doctoral dissertation, Univeritas Islam Negeri Alauddin Makassar.

[B54] Lacko H. S. (2011). *Examining Grey’s Anatomy: A Content Analysis of Elements of Medical School Communication Reform in a Popular Medical Drama*. Doctoral dissertation, Wake Forest University.

[B55] Lasekan O. (2021). Identification and adoption of themes in the big bang theory sitcom to foster academic cultural competencies of doctoral students in english for academic conversation classroom. *Front. Psychol*. 12:699662. 10.3389/fpsyg.2021.699662 PMC8458569 34566778

[B56] Lasley I. I. T. J. (1998). Paradigm shifts in the classroom. *Phi Delta Kappan* 80:84.

[B57] Latif M. M. (2016). *An Analysis of Characterization of The Main Characters in “The Social Network Movie Script”.* Sarjana Thesis, State Islamic Institute (IAIN) of Tulungagung.

[B58] Lewis L. Parsad B. Carey N. Bartfai N. Farris E. Smerdon B. (1999). *Teacher quality: a report on the preparation and qualifications of public school teachers.* Statistical Analysis Report. NCES 1999-080, Washington, DC: US Department of Education.

[B59] Luther A. (2000). The “old” method of teaching Vs. the “new” method of teaching. *J. Thought* 35 59–69.

[B60] Lynch T. A. (2019). *Examining teacher perceptions of the Stronge Teacher. Effectiveness Performance Evaluation System*. Ph.D. thesis, Glassboro, NJ: Rowan University.

[B62] Manganello J. Franzini A. Jordan A. (2008). Sampling television programs for content analysis of sex on TV: how many episodes are enough? *J. Sex Res.* 45 9–16. 10.1080/00224490701629514 18321026

[B63] Mariani L. (n.d.). *Images of teachers in Hollywood cinema (Part 3).* Available online at: http://www.cinemafocus.eu/Studi%20sul%20cinema/Teachers1firstpart.pdf (accessed September 9, 2021).

[B64] Mazandarani O. Troudi S. (2021). Measures and features of teacher effectiveness evaluation: perspectives from Iranian EFL lecturers. *Educ. Res. Policy Pract.* 1–24. 10.1007/s10671-021-09290-0

[B65] McBer H. (2000). *Research Into Teacher Effectiveness: A Model Of Teacher Effectiveness*. Research Report No. 216. London: Report by Hay McBer to the Department for Education and Employment (DfEE).

[B66] Miller P. (2012). *Ten Characteristics of a Good Teacher. In English Teaching Forum*, Vol. 50. Washington, DC: US Department of State. Bureau of Educational and Cultural Affairs, Office of English Language Programs, 36–38.

[B67] Murry J. P. Jr Dacin P. A. (1996). Cognitive moderators of negative-emotion effects: implications for understanding media context. *J. Consum. Res.* 22 439–447. 10.1086/209460

[B68] Neuman W. L. (2000). *Social Research Methods: Qualitative and Quantitative Approaches*, 4th Edn. Toronto, ON: Allyn & Bacon.

[B69] Nugent S. A. Shaunessy E. (2003). Using film in teacher training: viewing the gifted through different lenses. *Roeper Rev.* 25 128–134. 10.1080/02783190309554214

[B70] Park G. P. Lee H. W. (2006). The characteristics of effective English teachers as perceived by high school teachers and students in Korea. *Asia Pac. Educ. Rev.* 7 236–248. 10.1007/BF03031547

[B71] Paufler N. A. Sloat E. F. (2020). Using standards to evaluate accountability policy in context: school administrator and teacher perceptions of a teacher evaluation system. *Stud. Educ. Eval.* 64:100806. 10.1016/j.stueduc.2019.07.007

[B72] Perkmen S. Toy S. Caracuel A. Shelley M. (2018). Cross-cultural search for Big Five: development of a scale to compare personality traits of pre-service elementary school teachers in Turkey and Spain. *Asia Pac. Educ. Rev.* 19 459–468. 10.1007/s12564-018-9549-2

[B73] Pollard A. Tann S. (1987). *Reflective Teaching In The Primary School: A Handbook For Theclassroom.* London: Cassell.

[B74] Pouliot L. Cowen P. S. (2007). Does perceived realism really matter in media effects? *Media Psychol.* 9 241–259. 10.1080/15213260701285819

[B75] Raimo A. Devlin-Scherer R. Zinicola D. (2008). *Learning About Teachers Through Film. In the Educational Forum*, Vol. 66. London: Taylor & Francis Group, 314–323. 10.1080/00131720208984850

[B76] Resnick D. (2018). *Representing Education In Film: How Hollywood Portrays Educational Thought, Settings, And Issues.* New York, NY: Springer. 10.1057/978-1-137-59929-2

[B77] Robichaux R. R. Guarino A. J. (2012). The impact of implementing a portfolio assessment system on preservice teachers’ daily teaching reflections on improvement, performance and professionalism. *Creat. Educ.* 3 290–292. 10.4236/ce.2012.33045

[B78] Rockoff J. E. Jacob B. A. Kane T. J. Staiger D. O. (2011). Can you recognize an effective teacher when you recruit one? *Educ. Finance Policy* 6 43–74. 10.1162/EDFP_a_00022

[B79] Ryan P. A. Townsend J. S. (2010). Representations of teachers’ and students’ inquiry in 1950s television and film. *Educ. Stud.* 46 44–66. 10.1080/00131940903480258

[B80] Sandelowski M. (2000). Whatever happened to qualitative description? *Res. Nurs. Health* 23 334–340. 10.1002/1098-240X(200008)23:4<334::AID-NUR9>3.0.CO;2-G 10940958

[B81] Santiago P. Benavides F. (2009). “Teacher evaluation: a conceptual framework and examples of country practices”, *Paper presented at the OECD-Mexico Workshop “Towards a Teacher Evaluation Framework in Mexico: International Practices. Criteria and Mechanisms”*, Mexico City.

[B82] Schoenlank J. (2017). *School Administrators’ Perceptions of the James Stronge Teacher Evaluation System*. Dissertations and Theses (ETDs), Seton Hall University,

[B83] Schreier M. (2012). *Qualitative Content Analysis In Practice.* Thousand Oaks, CA: Sage publications.

[B84] Stronge J. H. (2012). Stronge teacher effectiveness performance evaluation system. Alexandria, VA: stronge & Associates. *Int. Biom. Soc.* 52 249–264.

[B85] Stronge J. H. (2018). *Qualities Of Effective Teachers.* Alexandria, VA: ASCD.

[B86] Stronge J. H. Richard H. B. Catano N. (2008). *Qualities Of Effective Principals.* Alexandria, VA: ASCD.

[B87] Stronge J. H. Ward T. J. Grant L. W. (2011). What makes good teachers good? A cross-case analysis of the connection between teacher effectiveness and student achievement. *J. Teach. Educ.* 62 339–355. 10.1177/0022487111404241

[B88] Stronge J. Tonneson V. C. (2012). *Teacher Effectiveness Performance Evaluation System Handbook. Retrieved December 23, 2020.* New Jersey, NJ: Stronge & Associates.

[B89] Superfine B. M. Gottlieb J. J. Smylie M. A. (2012). The expanding federal role in teacher workforce policy. *Educ. Policy* 26 58–78. 10.1177/0895904811435722

[B90] Tan C. (2006). Philosophical reflections from the silver screen: using films to promote reflection in preservice teachers. *Reflective Pract.* 7 483–497. 10.1080/14623940600987080

[B91] Taylor L. D. (2005). Effects of visual and verbal sexual television content and perceived realism on attitudes and beliefs. *J. Sex Res.* 42 130–137. 10.1080/00224490509552266 16123843

[B92] Van Beveren L. Rutten K. Vandermeersche G. Verdoodt I. (2018). Exploring educational taboos through school movies. A rhetorical analysis of student-teachers’ reflections. *Teach. Teach. Educ.* 75 187–198. 10.1016/j.tate.2018.06.008

[B93] Whitaker T. (2013). *What Great Teachers Do Differently: 17 Things That Matter Most.* New York, NY: Routledge.

[B94] Ye Y. Ward K. E. (2010). The depiction of illness and related matters in two top-ranked primetime network medical dramas in the United States: a content analysis. *J. Health Commun.* 15 555–570. 10.1080/10810730.2010.492564 20677058

[B95] Zarei L. Bagheri M. S. Sadighi F. Agosto V. (2019). Educational accountability in EFL contexts: providing remedies. *Cogent. Educ.* 6:1669381. 10.1080/2331186X.2019.1669381

[B96] Zeichner K. Liston D. (1987). Teaching student teachers to reflect. *Harv. Educ. Rev.* 57 23–49. 10.17763/haer.57.1.j18v7162275t1w3w

[B97] Zulfian Z. Sahril S. Omolu F. A. (2018). Teaching strategies in freedom writers movie. *J. Foreign Lang. Educ. Res.* 1 25–38.

