# Supplementary material for: Using Stronge Teacher Evaluation System to Assess the Effectiveness Level of Mr. Brown as an EFL Teacher in the Mind Your Language TV Show: An Attempt to Validate a Reflective Tool to Train Preservice EFL Teachers
Source: Front Psychol. 2021 Oct 6;12:648760. doi: 10.3389/fpsyg.2021.648760 (PMC8527878; doi:10.3389/fpsyg.2021.648760)
Supplement: Supplementary Table 1 — Assessment of Mr. Brown effectiveness as an EFL teacher using Stronge teacher evaluation system. [file Table_1.pdf]

Table 1: Assessment of Mr Brown Effectiveness as an EFL Teacher Using Stronge Teacher Evaluation System

N	Performance standard (Stronge, 2018).	Performance indicators	Evidences Show by Mr Brown	Manifest/ Latent	In-class/outclass	Scene description	Season, Episode Scene Time Counting
1	**Professional Knowledge:** The teacher demonstrates an appreciation of the curriculum, subject matter, and the formative needs of students by providing relevant learning experiences.	. • Demonstrates capacity to connect present content with past and future learning experiences,<br>• Showing of expertise in the subject matter.<br>•Considering learners' age group while developing instructional content and strategy.<br>• Communicates clearly by checking for clarity.<br>. Following curriculum standards.	Demonstrate his qualification and competency as an English teacher.	Manifest	out-class	The teachers' knowledge of English and qualification to teach is expressed when he told the Principal how impeccable is his teaching credential	Season 1, Episode 1,02:55-03:01
			Communicate clearly by checking for understanding and clarity	Manifest	In-class	Through repetition, especially while asking the students to sit down, and they did not understand. He has to make a gesture to establish communication	Season 1, Episode 1,05:25-05:32
			This lesson demonstrates an enriched curriculum	N/A	N/A	N/A	N/A
			Consider the age group of the students before creating an instructional strategy	Manifest	out-class	One of the tasks given to the students is a visitation to touristic places in England. This include cinema and zoo, Students are expected to narrate their experience in the classroom	Season 2, Episode 8, 01:45-09:00
2.	**Instructional Planning:** The teacher plans to use the school's curriculum, appropriate methods, tools, and<br>data to meet the needs of all students.	. Lesson Plan time is realistic for pacing and transitions.<br>. Lesson Plans is designed for differentiated instruction.<br>. Lesson plan is aligned to the objective of the curriculum of the school and student learning needs.<br>Creating a suitable long- and short-range lesson plans and adapt it when required	Lesson is paced	Manifest	In-class	Transition in the lesson plan is exemplified in how sentence development was taught. The lesson is well planned by asking them to define sentence, then develop it by asking students to use a different part of speech such as noun, adjective, verb and adverb to develop the sentence	Season 2, Episode 2, 06:20-08:40
			Lesson is aligned to curriculum standards	N/A	N/A	N/A	

			Lesson is planned before the class	Manifest	In-class	The teacher prepares for his class before the arrival of the students to the class. For example, the crossword game is drawn on the blackboard before the beginning of the class	Season 3, Episode 2, 07:16
			Lesson required critical thinking in order to understand concepts.	Manifest	In.class	Students are encouraged on critical thinking by asking them to offer suggestions on how to raise money for the school fete	Season 3, Episode 7, 05:45-8:35
			Limited differentiation of curriculum and syllabus	Manifest	In.class	The teacher justifies his adapting curriculum to the local inspector, which is functional English that helps students to understand the real-world application of English	Season 1, episode 1, 22:05-22:10
3	**Instructional Delivery:** The teacher promotes learning by utilizing an assortment of instructional techniques to meet students' needs.	-Engages students through active learning.<br>-Builds on learners' existing knowledge and skills.<br>-Differentiates instruction to fulfil the need of the students<br>-Reinforces learning goals consistently throughout lessons.<br>-Using several successful instructional strategies and resources.<br>-Uses instructional technology to improve student learning.<br>- Communication is clear, and understanding is checked.	Using of questioning technique	Manifest	In-class	The teacher lists different topics on the board. It ranges from Shakespeare, music, royal family, politics, British custom. The teacher asks a question based on the listed topic	Season 3, episode 3, 03:35-11:40
			Students are motivated to give feedback to each other on class activities	Manifest	In-class	The Italian student always help the other students in clarifying to whatever other students by explaining what the teacher is trying to say	Season 1, episode 2, 20:38-20:45
			Explaining concepts, and lesson content to students in a logical, sequential, and age-appropriate manner	Manifest	In-class	Explaining a concept of the salutation "SIR" in the British culture	Season 1, Episode 2, 18:40-18:49
			Other key teaching strategies and class topics	Manifest	In-class	Art of conversation	Season 3, episode 4, 03:10-06:45
						Pronunciation	Season 1, episode 2,04:42-05:05

						Essay	Season 1, episode 2
						Joke	Season 1, Episode 4
						Game	Season 2, Episode 7
						Debate	Season 2, Episode 4
						Using household items to teach vocabulary (shopping)	Season 1, Episode 4
						Impromptu speaking for one minute on a given topic	Season 1, episode 12. 02:19-09:01
			Creating an authentic learning environment to practice real-world application task	Manifest	In-class	The teacher asked the students in pairs to visit different places in England such as the zoo, TV stations so that they can practice their English	Season 3, Episode 5
			An excessive amount of time is devoted to an explanation at the beginning of each class.	Manifest	In-class	The teacher starts with a reading activity. The activity leads to the explanation of the figure of speech as a concept. This is followed by asking students to provide examples of the concept	Season 1, Episode 3, 14.30-16:55
			Using technology to promote learning n classroom.	Manifest	In-class	The teacher makes use of a tape recorder to teach pronunciation	Season 3, Episode 6, 06:30-10-15
4	**Assessment of/for Learning:** The instructor systematically utilizes allrelevant information to assess student academic progress, guide instructional content anddelivery methods and provide students with timely feedback.	-Student evaluation must be in with the established curriculum standards. -Uses assessment tools for both formative and summative purposes of informing and guide -Giving constructive and frequent feedback to students on their learning.	Providing feedback to students on their progress.	Manifest	In-class	The teacher has the habit of starting class by giving feedback on the previously assigned homework.	Season 1, Episode 10. 01.55-02:25
			Informing the students on the structure of the formative and summative examination	Manifest	In-class	Students were informed about the structure of the lower Cambridge certificate. The exam consist of oral, written, and dictation	Season 1, episode 11, 09:40-09:45
			Creating a standard protocol for the exam	Manifest	In-class	The teacher ensures that the students do not commit examination malpractices. By	Season 1, episode 11. 13:50-16:40

						disrupting the students plan to cheat at the exam	
			Use of formative assessment to vary instruction or pacing not evidenced.	Manifest	In-class	Using the mock test to prepare the student for the major exam	Season 1, episode 7,25:58-26:02
5	**Learning Environment:** The instructor uses tools, routines and procedures to provide an atmosphere that is respectful, constructive, secure, student-centered and conducive to learning.	-Arranges the classroom to improve learning by providing a conducive environment.<br>-Setting clear expectations, rules and regulations with student input, classroom rules and regulations, and enforces them consistently and fairly.<br>-Maximizes time for instruction by minimizing disruptions and distractions.<br>-Creating a climate of trust and teamwork by being fair, caring, respectful, and enthusiastic.<br>-Promoting cultural sensitivity and tolerance<br>-Respecting the diversity of, including language ethnicity, race, gender, and special needs.<br>-Promoting active listening and paying attention to students' needs and responses.<br>-Optimizing learning time by working with students individually and in small groups or whole groups.	Respects students' diversity, including language, culture, race, gender, and special need	Manifest	In-class	He respects his Japanese student by greeting him in a Japanese way by bowing to him as a form of exchanging pleasantry	Season 1, Episode 6. 05:28-05:30
			The classroom is configured to support multiple learning contexts (whole group, small group, and individual instruction).	Manifest	In-class	Students were asked to come to the front of the class to carry out a role play	Season 3, Episode 4, 02:68-06:45
			Maximizes instructional time by minimizing disruptions.	Manifest	In-class	The teacher maintain decorum in the class using bell	Season 1, episode 12.00:48-00:49
			Working with a small group who needed extra support.	Manifest	In-class	An inclusive class is run by giving extra attention to a newcomer to ensure what is said or taught in class is understood	Season 2, Episode 4. 06:30-06:48
			Promoting autonomous learning in classroom.	Manifest	In-class	Students are working on their class activity independently (autonomous learning)	Season 3, Episode 8. 01:35-01:37
			Having an excellent rapport with students by demonstrating caring and respect towards the students	Manifest	out-class	Relaxed class, students are happy with their teacher. Caring and assistance are exhibited when students are in trouble. For example, when Jamila in theft problem. He is always willing to solve the personal problem of the student	Season 1, Episode 5. 11:00-14:00
			Encouraging students to support each other during classroom activity.	Manifest	In-class	Students giving each other moral support through applause when one of them is making progress in her pronunciation	Season 1, Episode. 07:01-07:50

			Promoting cultural sensitivity by resolving conflict among his students	Manifest	In-class	He does not permit his students who belong to different religion Muslim and Sikh to be insulting each other	Season 1, episode 1, 22:01-22:10
			enforcing classroom rule	Manifest<br>Manifest	In-class<br>In-class	Several times the teachers enforced classroom regulations, for example, students are expected to speak only in English	Season 1, episode 3. 06:32-06:43
						The teacher used a handbell to get the students' attention and maintain decorum whenever the classroom is in rowdy session.	Season 1, episode 1. 04:43-04:44
6	**Professionalism:** The teacher is committed to professional integrity, communicates efficiently, takes responsibility for professional development and participates in it, resulting in better students' learning outcome.	-Collaborating within the school environment to facilitate students' wellbeing and success.<br>-Adhering to federal and state regulations, school policies, and ethical guidelines.<br>-Incorporating learning from professional development opportunities into instructional practice.<br>-Participating in professional development activities outside the classroom intended for school and student enhancement.<br>-Working in a collegial and other school staff in a collaborative manner<br>-Acts as a contributing member of the school's professional community learning group through collaboration with colleagues.	Demonstrate professionalism through dressing, speech, and in his relationship with the students.	Latent	In-class	The teacher is always dress up in a suit jacket. That explains his level of professionalism in a workplace. Regarding his relationship with the students, he is professional with the students.	In each episode
			Be a positive role model for all (English Department chair, leads workshops, participates on curriculum redesign committee).	N/A	N/A	N/A	N/A
			Collegial and collaborative (noted in English Department chair assignment letter).	Manifest	out-class	A strong professional relationship is built between Mr Brown and the Principal on how to improve the learning of the students	Season 3, Episode 6 . 01:40-01:45
			Participates in professional development (Differentiation PD).	N/A	N/A	N/A	N/A
			Ethical relationship with the students	Manifest	In-class	The teacher maintains an ethical relationship with his female student in the classroom.	Season 1, episode 2. 06:20-07:00

7	**Student Progress:** The teacher's job results in acceptable and measurable student academic progress. That is, the teacher's mandate to foster students' learning is fulfilled	-Sets periodic, acceptable, measurable, and appropriate achievement goals for student learning progress<br>-Provides evidence that achievement goals have been fulfilled.	Evidence of Student learning achievement	Latent	In-class	This is evidence when one of the student by the name Jamila could not speak in English in season 1, but by the end of season 2, she has been able to construct sentences in English	N/A
